# Immune Checkpoint Inhibitor Induced Pericarditis and Encephalitis in a Patient Treated With Ipilimumab and Nivolumab for Metastatic Melanoma: A Case Report and Review of the Literature

**DOI:** 10.3389/fonc.2021.749834

**Published:** 2021-12-09

**Authors:** Jorja Braden, Jenny H. Lee

**Affiliations:** ^1^ Department of Medical Oncology, Chris O’Brien Lifehouse, Sydney, NSW, Australia; ^2^ Department of Biomedical Sciences, Macquarie University, Sydney, NSW, Australia

**Keywords:** immunotherapy, melanoma, immune-related adverse effects, pericarditis, encephalitis, delayed immune reaction

## Abstract

Immune checkpoint inhibitors (ICIs) have dramatically improved outcomes in melanoma. Common ICI toxicities have become familiar to clinicians; however, rare delayed toxicities remain challenging given the paucity of data with such presentations. We present the unique case of a 61-year-old with metastatic melanoma with two rare, delayed ICI-induced toxicities. After resection of a large symptomatic parietal metastases, this patient received two doses of combination ipilimumab and nivolumab. Five weeks following his second dose, he developed ICI-induced pericarditis with associated pericardial effusion and early signs of tamponade. Corticosteroids were not administered due to a concurrent cerebral abscess. Administration of colchicine, ibuprofen, judicious monitoring, and cessation of immunotherapy led to the complete resolution of the effusion over several weeks. Seven months following his last dose of immunotherapy, the patient developed ICI-associated grade four autoimmune encephalitis, presenting as status epilepticus. High-dose steroid initiation led to rapid clinical improvement. The patient remains in near-complete response on imaging with no recurrence of pericardial effusion and partial resolution of neurological symptoms. ICI-induced pericardial disease and encephalitis carry substantial mortality rates and prompt diagnosis and management is critical. Clinicians must therefore remain vigilant for these rare toxicities regardless of duration of drug exposure or time since cessation of therapy.

## Introduction

The combination of immune checkpoint inhibitors (ICIs) ipilimumab and nivolumab have drastically improved outcomes for advanced melanoma patients with 5-year survival rates of 52% (1). This comes at the cost of increased rates of ICI-induced toxicities. The combination is associated with higher rates of a broad range of ICI-induced toxicities when compared to single-agent checkpoint inhibition, contributing to higher morbidity and mortality in these patients (1, 2). Severe ICI-induced pericarditis and encephalitis are exceedingly rare clinical entities accounting for far less than 1% of ICI toxicities and delayed events increase the rarity of such cases. With a paucity of data and a range of presentations, diagnosis and management remain a significant challenge. We report the case of a patient with two sequential rare ICI-associated toxicities of grade 3 pericarditis and grade 4 encephalitis presenting 1.5 months and 7.5 months after brief exposure to ipilimumab and nivolumab treatment for metastatic melanoma.

## Case Presentation

**Figure 1:**
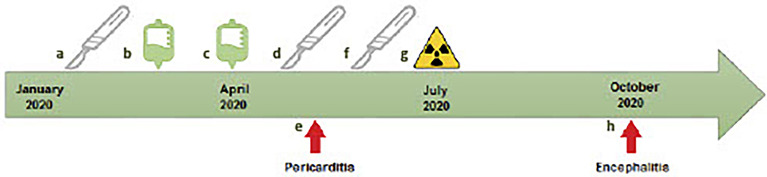
Timeline of case report of patient with rare delayed immune-related toxicities. **(A)** February 14, 2020, first brain metastasis resection. **(B)** March 2020, first cycle ipilimumab/nivolumab. **(C)** April 4, second cycle ipilimumab/nivolumab. **(D)** April 8, second brain metastasis resection. **(E)** May 12, presentation with immune-related pericarditis. **(F)** June 24, third brain metastasis resection. **(G)** July 14, stereotactic radiosurgery of resection cavity. **(H)** Presentation with auto-immune encephalitis.

A 61-year-old male was diagnosed with *de novo* metastatic melanoma in January 2020 after presenting with sudden onset left upper limb dyspraxia and confusion. Comorbidities included hemochromatosis and a distant history of meningococcal meningitis. Magnetic resonance imaging (MRI) brain demonstrated a large right parietal lesion. Computed tomography (CT) and positron emission tomography (PET) scan revealed left upper and lower lobe lung lesions, solitary liver lesion, and base of skull lesion. Histopathology confirmed BRAF/NRAS wild-type metastatic melanoma. He proceeded with resection of the right parietal lobe metastases in February followed by ipilimumab (3 mg/kg)/nivolumab (1 mg/kg) commencing in March (**Figure 1**).

MRI brain on the April 3 demonstrated intracranial recurrence with PET/CT confirming stable extracranial disease. A redo craniotomy was performed on April 8, complicated by the development of cerebral abscess and ventriculitis requiring burr hole and drainage. Cultures confirmed corynebacterium acnes and he commenced intravenous (IV) Cephalothin for a total of 12 weeks. Six weeks following his last dose of immunotherapy and while on IV antibiotics for his cerebral abscess, the patient developed severe peripheral edema, dyspnea, and tachycardia. Electrocardiograph (ECG) demonstrated sinus tachycardia, left axis deviation, and right bundle branch block. Transthoracic echocardiogram (TTE) revealed a new circumferential pericardial effusion with early signs of tamponade. Serial troponins remained normal, and cardiac MRI showed no evidence of myocarditis. A diagnosis of ICI-induced pericarditis with associated pericardial effusion was made. The patient was commenced on aggressive diuresis, colchicine 500 mcg daily and ibuprofen 500 mg three times daily. The active decision to withhold high-dose corticosteroids was made given the patient’s concomitant cerebral abscess. He was monitored with weekly echocardiograms by the treating cardiologist with gradual resolution of the pericardial effusion over 4 weeks. Immunotherapy was discontinued. In June 2020, the patient had a further recurrence of brain metastases. A third resection followed by stereotactic radiosurgery to the cavity were completed at that time.

**Figure 2:**
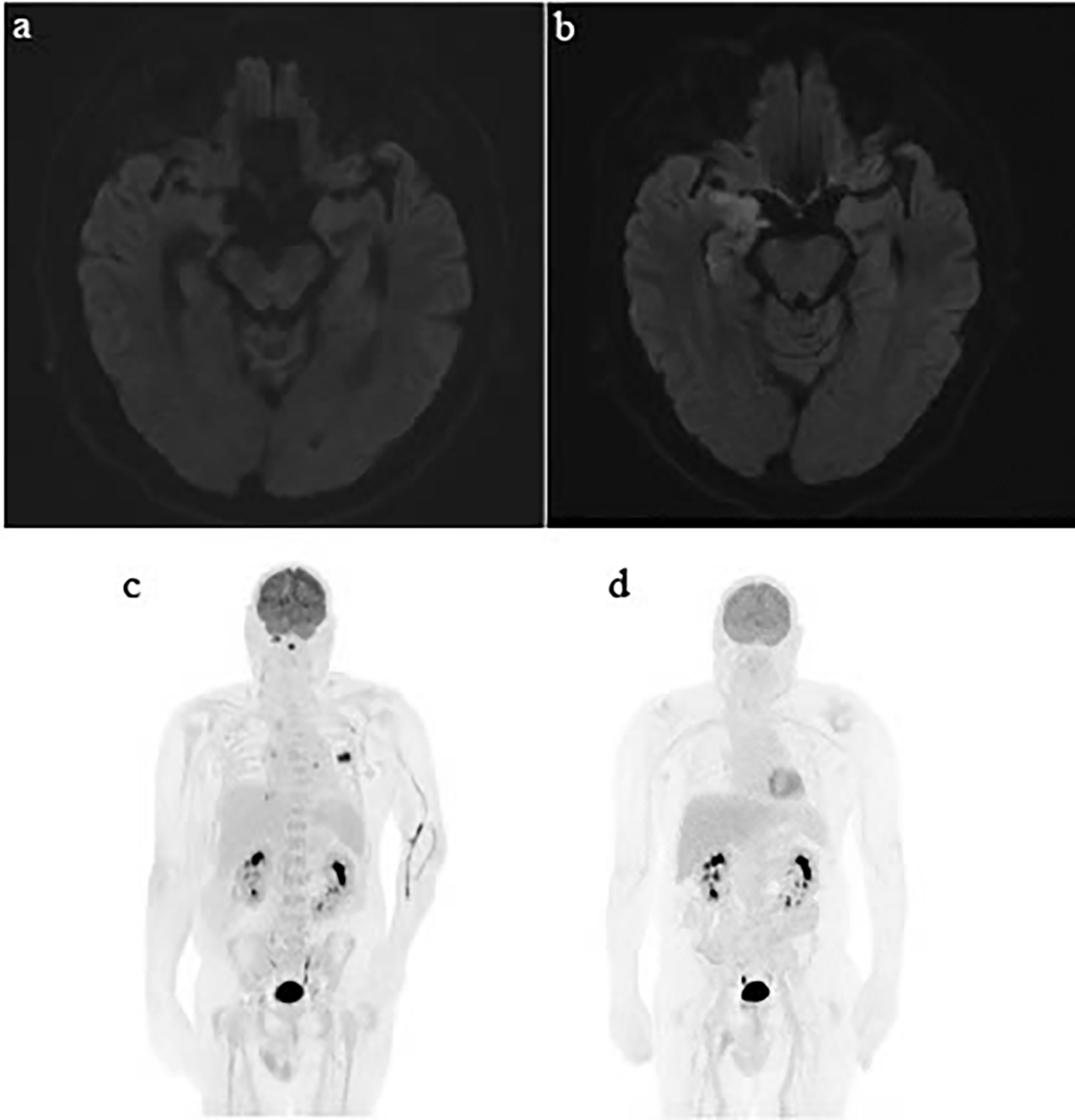
Serial MRI brain showing development of encephalitis and serial PET/CT demonstrating the patient’s durable response to immunotherapy. **(A)** MRI brain with gadolinium Sept 2, 2020—no abnormalities in medial temporal region. **(B)** MRI brain October 29, 2020 shows new T2/FLAIR hyperintensity in the right medial temporal lobes. **(C)** PET/CT March 2020. **(D)** PET CT March 2021.

Seven months following cessation of immunotherapy, the patient presented with sudden onset aphasia, left lower limb myoclonic jerks, and confusion. Further history revealed that the patient had developed subtle behavioral changes in the weeks prior. CT brain and angiogram showed no evidence of acute cerebrovascular event, infection, or intracranial disease progression. Laboratory results showed a normal CRP (0.7 m/L) and mild hyponatremia (129 mmol/L). An MRI brain revealed T2/FLAIR hyperintensity in the right mesotemporal lobe with differentials including encephalitis or postictal changes (**Figure 2**). Electroencephalogram (EEG) demonstrated lateralizing periodic discharges from the right temporal region. Empirical acyclovir was commenced following a lumbar puncture that demonstrated a mild elevation of protein 0.62 g/L, normal white cell count, negative bacterial/fungal cultures, and negative viral PCR panel. Despite up titration of antiepileptics, the patient continued to deteriorate with increasing confusion, fluctuating level of consciousness, persistent dysphasia, and development of visual hallucinations. Autoimmune encephalitis and antineuronal antibody panels were normal. ICI-induced encephalitis was considered the most likely diagnosis and methylprednisolone 500 mg IV/day was initiated, continued for 3 days, and followed by 2 days of 250 mg IV/day. There was a rapid and remarkable improvement in symptoms following steroid administration. A repeat EEG showed resolution of lateralizing periodic discharges from the right temporal region. He was discharged on 80 mg oral prednisone, which was slowly weaned over 2 months.

The patient has continued on surveillance since cessation of immunotherapy in April 2020. His most recent imaging in March 2021 demonstrated an ongoing near-complete response of his metastatic melanoma. His pericarditis has not recurred with significant but partial neurological recovery from his grade 4 encephalitis.

## Discussion

Immune-related adverse events (irAEs) remain a major challenge, contributing to morbidity and mortality for melanoma patients. Immune-related cardiac toxicity and neurologic toxicities account for a high proportion of fatal immune-related toxicities (2). For the majority of patients, these irAEs occur early; however, a minority of patients will develop irAEs late in treatment or following treatment cessation (2, 3). The definition of a delayed autoimmune adverse event (DIRE) is varied in literature. The majority of clinical trials define delayed safety adverse events as greater than 90 days after discontinuation of immunotherapy and thus this timeframe has been used in several recent reviews to define DIREs (3). A review by Couey et al. (3) included a collation of 194 trials and 367 case reports and only identified 25 DIREs, 2 pericarditis, and no encephalitis cases, highlighting the exceptional rarity of these cases.

Immune-related pericardial disease is rare and variably reported in the literature. The incidence of pericardial disease reported in a recent pharmacovigilance study was reported at 0.36% with combination anti-PD1 and anti-CTLA4 (4). Immune-related pericardial disease has a wide variation in both onset and presentation and in some instances may well be under-reported due to the variability in severity. Such variation may lead to delayed diagnosis and treatment. This is a significant concern due to the relatively high mortality rates associated with immune-related cardiac toxicity. ICI-induced pericarditis has a fatality rate of 13% (5). Further to this, pericardial disease may be associated with myocarditis, which carries a significantly higher mortality rate reported as high as 65.6% in combination immunotherapy (4). Taking into account variability in the literature, pericardial disease is most often seen early during treatment with the majority of patients developing cardiac toxicities within the first month of commencement of immunotherapy (4, 6). Patients with symptomatic pericardial disease often present with chest pain, signs and symptoms of right heart failure, or tamponade (7). Essential investigations include ECG, cardiac biomarkers, and echocardiogram. Cardiac MRI is a critical investigation to assess for myocarditis and should be completed where possible given the mortality rates associated with myocarditis (4, 5). Performance of pericardiocentesis is highly varied among institutions’ literature (5, 7) and should of course be balanced against the risks of this invasive procedure. Analysis of pericardial fluid can provide key diagnostic information. ICI-associated pericardial effusions commonly demonstrate a lymphocyte-rich infiltrate and the absence of malignant cells (5). As with most severe irAEs, high-dose corticosteroids are recommended; however, guidelines are based on limited case series. Cautiously selected patients may be suitable for management without high-dose corticosteroids *via* utilization of anti-inflammatories commonly employed for non-ICI-induced pericarditis, such as colchicine and ibuprofen. Such an approach is resource intensive as it requires close monitoring with serial TTEs and close cardiologist follow-up. On review of available literature, we identified two cases of ICI-induced pericarditis managed successfully without use of high-dose steroids. One case (5) was managed with therapeutic pericardiocentesis resulting in resolution of the effusion. The second case was successfully treated with colchicine and ibuprofen alone (8). This case highlights an approach that may be considered for patients with ICI-induced pericarditis where high dose steroids are contraindicated.

ICI-induced encephalitis is another extremely rare irAE with rates of 0.92% reported with combination immunotherapy (9, 10). ICI-induced encephalitis is reported to occur early during treatment with a median onset of 61 days reported in a large pharmacovigilance study by Johnson et al. (10). ICI-induced encephalitis typically presents with symptoms including altered mentation, speech disturbance, and altered level of consciousness (11). Diagnostic workup should be prioritized to exclude infectious etiologies. Diagnosis is often challenging given common overlapping toxicities. In this case, such overlapping toxicities included three cerebral metastasectomies, stereotactic radiotherapy, and a recent cerebral abscess. Key investigations for ICI-induced encephalitis include but are not limited to CT and MRI brain, EEG, LP with viral PCR and culture, autoimmune and paraneoplastic panels, serum inflammatory markers, and electrolytes (11). MRI changes typical of autoimmune encephalitis can include T2/FLAIR changes of the limbic system (11). CSF may show elevated white blood cell count and/or elevated protein levels. Prompt initiation of corticosteroids is crucial to decrease morbidity and mortality (12) in patients who develop ICI encephalitis with a mortality rate approaching 20% (2, 12).

Our case presented with ICI-induced encephalitis 7.5 months after cessation of immunotherapy. On review of the literature, we could identify only one other case of delayed ICI-induced encephalitis (13). Both cases were in patients who received treatment for prior brain metastases, responded rapidly to high-dose corticosteroids with partial neurological recovery in the short term. No medium- to long-term follow-up to assess ongoing neurological recovery was available for these cases.

## Conclusion

Combination immunotherapy has a wide range of potentially fatal immune-related toxicities with both ICI-induced pericarditis and ICI-induced encephalitis contributing to a high proportion of these fatalities (2). This case highlights the challenges clinicians face with life-threatening toxicity emerging many months after treatment cessation. As ipilimumab and nivolumab become more frequently employed and with an increasing population of long-term survivors, this case emphasizes the importance of constant vigilance for such toxicities. Ongoing collaboration and research are needed to produce robust guidelines to support clinicians in managing these rare presentations.

## Data Availability Statement

The original contributions presented in the study are included in the article/supplementary material. Further inquiries can be directed to the corresponding author.

## Ethics Statement

Ethical review and approval was not required for the study on human participants in accordance with the local legislation and institutional requirements. The patients/participants provided their written informed consent to participate in this study. Written informed consent was obtained from the individual(s) for the publication of any potentially identifiable images or data included in this article.

## Author Contributions

All authors listed have made a substantial, direct, and intellectual contribution to the work and approved it for publication.

## Conflict of Interest

JL is on the advisory board for Sanofi and has received honorarium from MSD, BMS, Novartis and travel support from BioRad.

The remaining author declares that the research was conducted in the absence of any commercial or financial relationships that could be construed as a potential conflict of interest.

## Publisher’s Note

All claims expressed in this article are solely those of the authors and do not necessarily represent those of their affiliated organizations, or those of the publisher, the editors and the reviewers. Any product that may be evaluated in this article, or claim that may be made by its manufacturer, is not guaranteed or endorsed by the publisher.
